# Supplementary material for: Modeling glioblastoma heterogeneity as a dynamic network of cell states
Source: Mol Syst Biol. 2021 Sep 16;17(9):e10105. doi: 10.15252/msb.202010105 (PMC8444284; doi:10.15252/msb.202010105)
Supplement: Supplementary file 6 — Source Data for Figure 5 [file MSB-17-e10105-s004.zip › Figure5A_sourcedata/GSEA_3017/hallmarks_stateF.GseaPreranked.1621934628066/HALLMARK_COAGULATION.html]

Details for gene set HALLMARK\_COAGULATION[GSEA]

|  || Dataset | state33017 |
| Phenotype | NoPhenotypeAvailable |
| Upregulated in class | na\_pos |
| GeneSet | HALLMARK\_COAGULATION |
| Enrichment Score (ES) | 0.46686587 |
| Normalized Enrichment Score (NES) | 2.0039322 |
| Nominal p-value | 0.0 |
| FDR q-value | 0.0061131553 |
| FWER p-Value | 0.053 |
Table: GSEA Results Summary

  

Fig 1: Enrichment plot: HALLMARK\_COAGULATION      
 Profile of the Running ES Score & Positions of GeneSet Members on the Rank Ordered List

  

| PROBE | GENE SYMBOL | GENE\_TITLE | RANK IN GENE LIST | RANK METRIC SCORE | RUNNING ES | CORE ENRICHMENT || 1 | CLU |  |  | 5 | 1.852 | 0.1209 | Yes |
| 2 | F3 |  |  | 41 | 0.978 | 0.1702 | Yes |
| 3 | A2M |  |  | 42 | 0.974 | 0.2349 | Yes |
| 4 | SPARC |  |  | 52 | 0.929 | 0.2926 | Yes |
| 5 | LRP1 |  |  | 92 | 0.765 | 0.3259 | Yes |
| 6 | PROS1 |  |  | 123 | 0.708 | 0.3595 | Yes |
| 7 | HTRA1 |  |  | 229 | 0.568 | 0.3502 | Yes |
| 8 | C1R |  |  | 249 | 0.554 | 0.3785 | Yes |
| 9 | S100A13 |  |  | 261 | 0.544 | 0.4097 | Yes |
| 10 | LAMP2 |  |  | 308 | 0.506 | 0.4227 | Yes |
| 11 | ISCU |  |  | 325 | 0.498 | 0.4486 | Yes |
| 12 | MMP2 |  |  | 357 | 0.485 | 0.4669 | Yes |
| 13 | TIMP3 |  |  | 516 | 0.428 | 0.4244 | No |
| 14 | CTSO |  |  | 584 | 0.403 | 0.4211 | No |
| 15 | ADAM9 |  |  | 722 | 0.365 | 0.3839 | No |
| 16 | P2RY1 |  |  | 939 | 0.319 | 0.3082 | No |
| 17 | SIRT2 |  |  | 1018 | 0.307 | 0.2936 | No |
| 18 | CD9 |  |  | 1070 | 0.297 | 0.2904 | No |
| 19 | C1S |  |  | 1087 | 0.294 | 0.3028 | No |
| 20 | LGMN |  |  | 1110 | 0.291 | 0.3123 | No |
| 21 | FN1 |  |  | 1238 | 0.275 | 0.2736 | No |
| 22 | CAPN5 |  |  | 1339 | 0.263 | 0.2462 | No |
| 23 | ITGA2 |  |  | 1395 | 0.257 | 0.2385 | No |
| 24 | PRSS23 |  |  | 1690 | -0.316 | 0.1276 | No |
| 25 | SERPINE1 |  |  | 2161 | -0.843 | -0.0274 | No |
| 26 | TFPI2 |  |  | 2209 | -1.026 | 0.0197 | No |
Table: GSEA details [plain text format]

  

Fig 2: HALLMARK\_COAGULATION: Random ES distribution      
 Gene set null distribution of ES for **HALLMARK\_COAGULATION**
